# Supplementary material for: Systematic review protocol of aetiology of mechanical bowel obstruction in low-and-middle income countries: Has anything changed in the last two decades?
Source: PLoS One. 2024 May 9;19(5):e0295477. doi: 10.1371/journal.pone.0295477 (PMC11081211; doi:10.1371/journal.pone.0295477)
Supplement: S1 Checklist — (DOC) [file pone.0295477.s001.doc]

**PRISMA-P (Preferred Reporting Items for Systematic review and Meta-Analysis Protocols) 2015 checklist: recommended items to address in a systematic review protocol***

| Section and topic | Item No | Checklist item |
| --- | --- | --- |
| ADMINISTRATIVE INFORMATION | | |
| Title: |  |  |
| Identification | 1a | Identify the report as a protocol of a systematic review  Systematic Review Protocol of aetiology of mechanical bowel obstruction in Low-and-middle income countries: Has anything changed in the last two decades? |
| Update | 1b | If the protocol is for an update of a previous systematic review, identify as such  No |
| Registration | 2 | If registered, provide the name of the registry (such as PROSPERO) and registration number  PROSPERO CRD42023468901 |
| Authors: |  |  |
| Contact | 3a | Provide name, institutional affiliation, e-mail address of all protocol authors; provide physical mailing address of corresponding author  Yakubu Kevin Kwarshak^1*^, Mohammed Nakodi Yisa^2^, Oghenegare Asheaba Kigbu^3^, Daniel Akut John^4^, Nankam David Jimwan^5^, Karen Chineme Ubabuike^6^, Peter Mkurtar Yawe^7^  [kwarshak87.kk@gmail.com](mailto:kwarshak87.kk@gmail.com)  Department of Surgery, Division of Urology, Jos University Teaching Hospital, JUTH, Plateau state, Nigeria. |
| Contributions | 3b | Describe contributions of protocol authors and identify the guarantor of the review  Yakubu Kevin Kwarshak: Conceptualization, literature search, data synthesis, mauscript drafting and editing,  Mohammed Nakodi Yisa: literature searching, data synthesis, manuscript drafting and editing  Oghenegare Asheaba Kigbu: Data extarction, synthesis and manuscripting editing  Daniel Akut John: Data extarction, synthesis and manuscripting editing  Nankam David Jimwan: Data extraction, synthesis and manuscripting editing  Karen Chineme Ubabuike: Data extraction, synthesis and manuscripting editing  Peter Mkurtar Yawe: Data extarction, synthesis and manuscripting editing  Validation of manuscript: All Authors |
| Amendments | 4 | If the protocol represents an amendment of a previously completed or published protocol, identify as such and list changes; otherwise, state plan for documenting important protocol amendments  No |
| Support: |  |  |
| Sources | 5a | Indicate sources of financial or other support for the review  No funding |
| Sponsor | 5b | Provide name for the review funder and/or sponsor  Not applicable |
| Role of sponsor or funder | 5c | Describe roles of funder(s), sponsor(s), and/or institution(s), if any, in developing the protocol  Not applicable |
| INTRODUCTION | | |
| Rationale | 6 | Describe the rationale for the review in the context of what is already known  To the best of our knowledge, no systematic review has been conducted in all LMICs to assess the aetiological pattern of mechanical bowel obstruction, leaving a lack of up-to-date evidence to guide surgical trainees and surgeons in the prompt diagnosis and management of such an important surgical emergency. |
| Objectives | 7 | Provide an explicit statement of the question(s) the review will address with reference to participants, interventions, comparators, and outcomes (PICO)  Therefore, to fill this knowledge gap, this study aims to systematically review the existing literature on the aetiology of mechanical bowel obstruction with a focus on understanding the most common cause of mechanical bowel obstruction in LMICs in both paediatric and adult populations to guide surgical practice |
| METHODS | | |
| Eligibility criteria | 8 | Specify the study characteristics (such as PICO, study design, setting, time frame) and report characteristics (such as years considered, language, publication status) to be used as criteria for eligibility for the review  We will consider studies published between 2002 and 2022 in low- and middle-income countries (LMICs) that are either case series, case-control or cohort studies reporting on the aetiology of mechanical small bowel obstruction in any age group. Studies must be published in either English or French. We will exclude animal studies, studies with different designs, those published in high-income countries, those outside the specified date range, or those published in languages other than English or French |
| Information sources | 9 | Describe all intended information sources (such as electronic databases, contact with study authors, trial registers or other grey literature sources) with planned dates of coverage  We will conduct a literature search using Ovid MEDLINE, Ovid Embase, CINAHL on EBSCO and Web of Science databases. In addition, we will manually search the reference lists of the included studies to find additional relevant articles. |
| Search strategy | 10 | Present draft of search strategy to be used for at least one electronic database, including planned limits, such that it could be repeated  A search strategy was developed using the key terms 'small bowel obstruction', 'lower-middle-income countries' and 'aetiology'. The MEDLINE search strategy, adapted for other databases, is shown in Figure 1. |
| Study records: |  |  |
| Data management | 11a | Describe the mechanism(s) that will be used to manage records and data throughout the review  A data extraction form adapted from the JBI will be used for data extraction |
| Selection process | 11b | State the process that will be used for selecting studies (such as two independent reviewers) through each phase of the review (that is, screening, eligibility and inclusion in meta-analysis)  Data extraction will be carried out by two reviewers; reviewer A will extract the data, while reviewer B will check the accuracy of the extracted data |
| Data collection process | 11c | Describe planned method of extracting data from reports (such as piloting forms, done independently, in duplicate), any processes for obtaining and confirming data from investigators  The data extraction form will be pre-tested independently by two reviewers using randomly selected articles |
| Data items | 12 |  |
| Outcomes and prioritization | 13 | List and define all outcomes for which data will be sought, including prioritization of main and additional outcomes, with rationale  Author name(s), Year of publication, study design, country of publication, age range of study population, sample size and reported causes of mechanical bowel obstruction |
| Risk of bias in individual studies | 14 | Describe anticipated methods for assessing risk of bias of individual studies, including whether this will be done at the outcome or study level, or both; state how this information will be used in data synthesis  We will use the Joanna Briggs Institute (JBI) tools for each study design (case series, case-control, cohort) to be included in the review |
| Data synthesis | 15a | Describe criteria under which study data will be quantitatively synthesised  We anticipate that the eligible studies will be quite heterogeneous in terms of their design, outcomes of interest, populations and comorbidities. Therefore, results may be synthesised descriptively without meta-analysis. |
|  | 15b | If data are appropriate for quantitative synthesis, describe planned summary measures, methods of handling data and methods of combining data from studies, including any planned exploration of consistency (such as I^2^, Kendall’s τ)  Histogram |
|  | 15c | Describe any proposed additional analyses (such as sensitivity or subgroup analyses, meta-regression)  Sub-group analysis based on age and WHO regions |
|  | 15d | If quantitative synthesis is not appropriate, describe the type of summary planned  Graphs, charts and tables |
| Meta-bias(es) | 16 | Specify any planned assessment of meta-bias(es) (such as publication bias across studies, selective reporting within studies) |
|  |  | None |
| Confidence in cumulative evidence | 17 | Describe how the strength of the body of evidence will be assessed (such as GRADE)  GRADE |

*** It is strongly recommended that this checklist be read in conjunction with the PRISMA-P Explanation and Elaboration (cite when available) for important clarification on the items. Amendments to a review protocol should be tracked and dated. The copyright for PRISMA-P (including checklist) is held by the PRISMA-P Group and is distributed under a Creative Commons Attribution Licence 4.0.**

*From: Shamseer L, Moher D, Clarke M, Ghersi D, Liberati A, Petticrew M, Shekelle P, Stewart L, PRISMA-P Group. Preferred reporting items for systematic review and meta-analysis protocols (PRISMA-P) 2015: elaboration and explanation. BMJ. 2015 Jan 2;349(jan02 1):g7647.*
